# Prevalence and trends for Aboriginal and Torres Strait Islander children living with cerebral palsy: A birds‐eye view

**DOI:** 10.1111/dmcn.15617

**Published:** 2023-05-05

**Authors:** Tanya Martin, Sarah McIntyre, Emma Waight, Gareth Baynam, Linda Watson, Katherine Langdon, Susan Woolfenden, Hayley Smithers‐Sheedy, Juanita Sherwood, ACPR Birds‐Eye View Group

**Affiliations:** ^1^ School of Nursing and Midwifery The University of Sydney Camperdown New South Wales Australia; ^2^ Cerebral Palsy Alliance/Research Institute, Specialty of Child & Adolescent Health The University of Sydney Camperdown New South Wales Australia; ^3^ Department of Health, Western Australian Register of Developmental Anomalies Government of Western Australia Perth Western Australia Australia; ^4^ Perth Children's Hospital Perth Western Australia Australia; ^5^ Discipline of Paediatrics, School of Clinical Medicine UNSW Sydney Sydney New South Wales Australia; ^6^ Sydney Institute of Women, Children and their Families Sydney Local Health District Sydney New South Wales Australia; ^7^ Jumbunna Institute for Indigenous Education and Research University of Technology Sydney Sydney New South Wales Australia

## Abstract

**Aim:**

To provide a birds‐eye view of the trends of cerebral palsy (CP) for Australian Aboriginal and Torres Strait Islander children and young adults.

**Method:**

Data were obtained for this population‐based observational study from the Australian Cerebral Palsy Register (ACPR), birth years 1995 to 2014. The Indigenous status of children was classified by maternal Aboriginal and Torres Strait Islander or non‐Indigenous status. Descriptive statistics were calculated for socio‐demographic and clinical characteristics. Prenatal/perinatal and post‐neonatal birth prevalence was calculated per 1000 live births and per 10 000 live births respectively, and Poisson regression used to assess trends.

**Results:**

Data from the ACPR were available for 514 Aboriginal and Torres Strait Islander individuals with CP. Most children could walk independently (56%) and lived in urban or regional areas (72%). One in five children lived in socioeconomically disadvantaged remote/very remote areas. The birth prevalence of prenatal/perinatal CP declined after the mid‐2000s from a high of 4.8 (95% confidence interval 3.2–7.0) to 1.9 per 1000 live births (95% confidence interval 1.1–3.2) (2013–2014), with marked declines observed for term births and teenage mothers.

**Interpretation:**

The birth prevalence of CP in Aboriginal and Torres Strait Islander children in Australia declined between the mid‐2000s and 2013 to 2014. This birds‐eye view provides key stakeholders with new knowledge to advocate for sustainable funding for accessible, culturally safe, antenatal and CP services.

**What this paper adds:**

- Birth prevalence of cerebral palsy (CP) is beginning to decline for Aboriginal and Torres Strait Islanders.
- Recent CP birth prevalence for Aboriginal and Torres Strait Islanders is 1.9 per 1000 live births.
- Most children with CP live in more populated areas rather than remote or very remote areas.
- One in five Aboriginal and Torres Strait Islander children with CP live in socioeconomically disadvantaged remote areas.

## What this paper adds

This paper provides an overview of the prevalence and trends for Aboriginal and Torres Strait Islander children living with cerebral palsy (1995–2014).

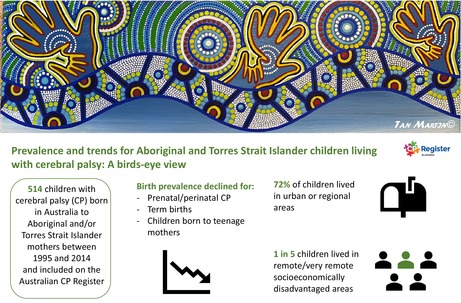

This original article is commented on by Lynch on pages 1417–1418 of this issue.

There is a letter to the editor on this original article by Rowse on page 1530.

**Abbreviations**
ACPR: Australian Cerebral Palsy Register
ARIA: Accessibility Remoteness Index of Australia
SEIFA: Socio‐Economic Indexes for Areas

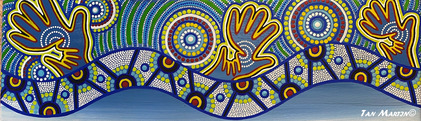

*Artwork: A Bird's‐Eye View of Aboriginal inclusion and knowledge sharing within the Australian Cerebral Palsy Register (ACPR) by first author Tanya Martin*.

*The ACPR Group would like to acknowledge the traditional owners of all the lands on which this work has been conducted. We pay our respects to the children, their families, and their elders past and present – for they are the ones that hold the memories, the stories, the traditions, and the cultures of Aboriginal and Torres Strait Islander people across the lands, that make up this continent*.

Aboriginal and Torres Strait Islander peoples are a proud, linguistically, and culturally diverse population that represents 3.2% of the total Australian population.^1^ The Aboriginal and Torres Strait Islander population is growing and predicted to grow by 136% by 2050.^2^ One in five Aboriginal and Torres Strait Islander people live in remote and very remote settings, where they constitute up to 90% of residents.^3^

However, because of the continuing legacy of colonization, dispossession of land, disruption, and censoring of language and cultural practices (a violation of international human rights [Mabo Decision 1992]),^4^ Aboriginal and Torres Strait Islander people experience socioeconomic, criminal justice, health, and disability inequities compared with non‐Indigenous Australians.^2^, ^5^, ^6^ For many generations, a deficit‐based research, service, and policy discourse has dominated the health and well‐being narratives of Aboriginal and Torres Strait Islander people. Inequities caused by policy based on these narratives have been further compounded by a shifting of blame for these inequities to Aboriginal and Torres Strait Islanders themselves.^7^, ^8^

Despite these injustices,^9^ Aboriginal and Torres Strait Islander peoples remain resilient.^10^ Contemporary epidemiological research must reflect this resilience and address these inequities. When led by Aboriginal and Torres Strait Islander people, it has the potential to simultaneously harness epidemiology's persuasive power in public health and policy to promote equity, while also critically challenging the legacy of colonialist misrepresentation of Indigenous health in population statistics and qualitative discourse.^11^

There is a lack of epidemiological research into cerebral palsy (CP) for Australian Aboriginal and Torres Strait Islander children. CP is a lifelong condition and people with lived experience describe varying degrees of difficulty with muscle coordination, muscle control, muscle tone, reflexes, balance, and posture. In Australia, CP is seen across all socioeconomic groups; however, socioeconomic disadvantage is associated with more severe impairment and subsequently greater health‐service needs.^12^ In the only other Australian epidemiological study, Blair et al. reported that Aboriginal and Torres Strait Islander children had a higher birth prevalence of CP (particularly post‐neonatally acquired) than non‐Indigenous children, which was based on birth years 1996 to 2005.^13^ However, the research did not include an examination of socioeconomic status and area of residence in the context of service accessibility and did not report birth prevalence trends. In the intervening years, there has been a significant decline in the birth prevalence of CP in Australia.^14^, ^15^ It is yet to be determined whether CP birth prevalence has declined for Aboriginal and Torres Strait Islander children.

The purpose of this study was twofold: (1) to provide estimates of socio‐demographic and clinical profiles of Aboriginal and Torres Strait Islander children with CP; (2) to provide prevalence estimates and assess trends in birth prevalence of CP (term and preterm, and in teenage mothers) for Aboriginal and Torres Strait Islander peoples (birth years 1995–2014).

## METHOD

In conducting this research, which was led by Aboriginal researchers, an Indigenous lens and a strengths‐based perspective was used.^7^, ^16^ The Community, Aboriginal and Torres Strait Islander Reference Group was developed to facilitate Aboriginal and Torres Strait Islander and non‐Indigenous consumer involvement in the use and reporting of Australian Cerebral Palsy Register (ACPR) data in research. Development of the study and interpretation of the findings were generated from a reciprocal balance between Aboriginal and Torres Strait Islander peoples', clinicians'/researchers', and people with CP and their families' experience and knowledge through yarning. Yarning is an important cultural form of communication for Aboriginal and Torres Strait Islander people; it refers to the sharing of information and stories, and it can set expectations and determine accountability.^17^

### Study cohort

In this population‐based observational study we used numerator data from the ACPR, which holds non‐identifiable data that are collected every 2 years from each of the eight Australian states and territories. These registers collect an agreed minimum data set for children with CP born in their jurisdiction.^18^ Data are confirmed when the child is around 5 years of age either from their medical record (on the basis of clinical assessment) or from a clinical assessment undertaken by staff specifically for the CP register.^19^ If a child passes away before their fifth birthday, and their CP diagnosis had been confirmed, their data are still included.

For all descriptive analyses, data from all state and territory CP registers were included (birth years 1995–2014). For the analysis of birth prevalence, numerator data for birth years 1995 to 2014 were included from three long‐standing registers (South Australia, Victoria, and Western Australia) where the researchers were confident that all or very nearly all children with CP were identified across the whole period at the time of data extraction (July 2020).^18^

### Study variables

Birth years were aggregated and reported by 10 2‐year epochs. Maternal Aboriginal and Torres Strait Islander status was described as Aboriginal and/or Torres Strait Islander (Aboriginal but not Torres Strait Islander, Torres Strait Islander but not Aboriginal, Aboriginal and Torres Strait Islander) and non‐Indigenous.^20^

Neighbourhood socioeconomic status was estimated by taking birth postcode or, if unavailable, postcode at age 5 years and linking this to the related Socio‐Economic Indexes for Areas (SEIFA) Indices of Relative Socioeconomic Advantage and Disadvantage deciles published by the Australian Bureau of Statistics.^21^ These SEIFA Indices deciles were then combined into quintiles, with quintile 1 being the most disadvantaged and quintile 5 being the most advantaged.

Residential postcode at birth (or at age 5 years if residential postcode at birth were missing) was used to determine accessibility and remoteness by applying the Accessibility Remoteness Index of Australia+2016 (ARIA+ 2016). ARIA+ 2016 data were sourced from the Hugo Centre for Population and Housing, University of Adelaide.^22^ ARIA+ 2016 data were categorized by major city, inner regional, outer regional, remote, and very remote.

Maternal age was reported as younger than 20 years, 20 to 34 years, or 35 years or more. Sex was reported as male or female; gestational age as less than 28 weeks, 28 to 31 weeks, 32 to 36 weeks, and 37 weeks or more; and birthweight as less than 1000 g, 1000 g to 1499 g, 1500 g to 1999 g, 2000 g to 2499 g, 2500 g to 2999 g, or 3000 g or more. Small for gestational age was defined as birthweight below the 10th centile for gestational age, and sex using Australian birthweight centiles for singleton births.^23^ Timing of brain injury or insult was described as prenatal/perinatal (occurring during pregnancy or up to 28 days after birth) or post‐neonatal (brain injury acquired more than 28 days after birth and before 2 years of age). The following variables were confirmed at 5 years of age by combined specialist assessments: predominant motor type (spastic unilateral [monoplegia, hemiplegia], spastic bilateral [diplegia, triplegia, quadriplegia], ataxic, dyskinetic, hypotonic), Gross Motor Function Classification System (levels I–II [independent mobility], levels III–V [mobility device required]),^24^ intellectual impairment (no/borderline/mild, moderate–severe), speech (no/some impairment, non‐verbal), epilepsy (no active epilepsy, epilepsy), hearing (no/some impairment, deaf), and vision (no/mild impairment, functionally blind).

Denominator data (live births born 1995–2014) were sourced from the National Perinatal Data Collection, Australian Institute of Health and Welfare.^25^

### Statistical analyses

Descriptive statistics (counts and percentages) were calculated for demographic and clinical characteristics of CP. If a state or territory had over 20% missing data for a variable, then that state or territory's data were excluded from the analysis for that variable. Prenatal/perinatal and post‐neonatal CP birth prevalences were calculated per 1000 live births and per 10 000 live births respectively, both with 95% confidence intervals. The numerators were the count of eligible children (Aboriginal and/or Torres Strait Islander or Non‐Aboriginal and/or Torres Strait Islander) with either prenatal/perinatal or post‐neonatally acquired CP (as described above) born in states with CP registers with total population ascertainment (South Australia, Victoria, and Western Australia), for each epoch and included in these registers. The denominator was a count of all live births born in these states during each 2‐year epoch. Birth prevalence for each epoch was calculated as the ratio of the numerator to denominator expressed as the number of cases per 1000 live births for prenatal/perinatal CP and the number of cases per 10 000 live births for post‐neonatally acquired CP. Two‐year moving averages were used for graphical representation of the data. Poisson regression was used to assess temporal trends, with the count as the outcome variable, including an offset term for the denominator to account for differences in denominators across state registers. Birth year was used as the predictor variable to assess whether there was a significant trend over time.

### Ethics

Ethical oversite for the ACPR (2020/463) is held by The University of Sydney Human Research Ethics Committee and the Aboriginal Health and Medical Research Council (1388/18). Additional specific research approvals were received from the Aboriginal Health and Medical Research Council (1623/20) and individual State and Territories *–* Human Research Ethics Committee of the Northern Territory Department of Health and Menzies School of Health Research (2020–3612), Aboriginal Health Research Ethics Committee (South Australia) (04–20‐872), Western Australian Aboriginal Health Ethics Committee (975), and Women's and Children's Health Network Research and Ethics Committee (South Australia) (2020/GEM02413).

## RESULTS

In the birth years 1995 to 2014 there were 514 Aboriginal and Torres Strait Islander children and young adults with CP included on the ACPR. Of these, 11% had CP resulting from a post‐neonatal injury. More than half of all children were male (55%) and two‐thirds of those with post‐neonatal CP were male. Ninety‐one per cent of children were born a singleton, with 9% a twin or triplet. Half of the children born a singleton were within the typical birthweight range, with 85% born at an appropriate weight for gestational age.

One‐third of all children had spastic unilateral CP and one‐half had spastic bilateral CP as their predominant motor type. Children with post‐neonatal CP only rarely had a non‐spastic predominant motor type. Most children and young people could walk without requiring aids (GMFCS levels I and II) (56%), had either no intellectual impairment or a mild intellectual impairment (72%), could talk (69%), did not have active epilepsy (59%), and had functional hearing (96%) and vision (91%) (Table 1).

**TABLE 1:** Aboriginal and/or Torres Strait Islander children with cerebral palsy, birth years 1995–2014.

All states and territories	Prenatal/perinatal, *n* (%)^a^	Post‐neonatal, *n* (%)^a^	All cerebral palsy, *n* (%)^a^
Total	457	57	514
Sex			
Female	208 (45.5)	20 (35.1)	228 (44.4)
Male	249 (54.5)	37 (64.9)	286 (55.6)
Unknown	—	—	—
Plurality			
Singletons	379 (90.7)	49 (90.7)	428 (90.7)
Twins/triplets	39 (9.3)	5 (9.3)	44 (9.3)
Unknown	39 (8.5)	3 (5.2)	42 (8.2)
Gestational age			
<28 weeks	57 (13.5)	—	57 (12.1)
28–31 weeks	61 (14.4)	^b^	63 (13.3)
32–36 weeks	77 (18.2)	10 (20.4)	87 (18.4)
≥37 weeks	228 (53.9)	37 (75.5)	265 (56.1)
Unknown	34 (7.4)	8 (14.0)	42 (8.2)
Birthweight (g)^b^			
<1000	42 (14.0)	—	42 (12.1)
1000–1499	31 (10.3)	^b^	32 (9.2)
1500–1999	28 (9.3)	^b^	30 (8.7)
2000–2499	44 (14.6)	7 (15.6)	51 (14.7)
2500–2999	54 (17.9)	13 (28.9)	67 (19.4)
≥3000^c^	102 (33.9)	22 (48.9)	124 (35.8)
Unknown	21 (6.5)	5 (10.0)	26 (7.0)
Small for gestational age (singletons only)^b^			
<28 weeks	6 (18.2)	—	6 (18.2)
28–31 weeks	4 (11.1)	—	4 (10.8)
32–36 weeks	7 (15.6)	—	7 (13.7)
≥37^c^ weeks	46 (30.7)	7 (15.9)	53 (28.8)
Unknown	11 (4.2)	1 (2.2)	12 (3.8)
Predominant motor type			
Spastic unilateral	141 (32.8)	26 (49.1)	167 (34.6)
Spastic bilateral	227 (52.7)	24 (45.4)	251 (52.0)
Diplegia	118 (27.4)	7 (13.2)	125 (25.9)
Tri/quadriplegia	109 (25.3)	17 (32.2)	126 (26.1)
Ataxic	14 (3.3)	—	14 (2.9)
Dyskinetic	31 (7.2)	3 (5.7)	34 (7.0)
Hypotonic	17 (4.0)	—	17 (3.5)
Unknown	27 (6.0)	4 (7.0)	31 (6.0)
Gross Motor Function Classification System level^c^			
I–II	238 (56.9)	30 (54.5)	267 (56.7)
III–V	180 (43.1)	25 (45.4)	205 (43.4)
Unknown	27 (6.1)	2 (3.6)	29 (5.8)
Intellectual impairment			
No, borderline, or mild impairment	304 (72.7)	32 (62.7)	336 (71.6)
Moderate–severe impairment	114 (27.3)	19 (37.3)	133 (28.4)
Unknown	39 (8.5)	6 (10.5)	45 (8.8)
Speech^c^			
No or mild impairment	281 (69.6)	33 (66.0)	314 (69.2)
Non‐verbal	123 (30.4)	17 (34.0)	140 (30.8)
Unknown	41 (9.2)	6 (10.7)	47 (9.4)
Epilepsy^c^			
No active epilepsy	245 (60.0)	27 (52.9)	272 (59.3)
Epilepsy	163 (40.0)	24 (47.1)	186 (40.7)
Unknown	38 (8.5)	5 (9.0)	43 (8.6)
Hearing^c^			
No or mild impairment	373 (96.4)	46 (93.9)	419 (96.1)
Deaf	14 (3.6)	^b^	17 (3.9)
Unknown	58 (13.0)	7 (12.5)	65 (13.0)
Vision^c^			
No or mild impairment	368 (92.2)	43 (84.3)	411 (91.3)
Functionally blind	31 (7.8)	8 (15.7)	39 (8.7)
Unknown	46 (10.3)	5 (8.8)	51 (9.2)

^a^ Percentage calculated by *n*/total *n* minus unknown *n*.

^b^ Excludes data for New South Wales/Australian Capital Territory.

^c^ Excludes South Australia children who had not received a follow‐up assessment at the time of data provision.

^d^ Fewer than five in the cell.

**FIGURE 1:**
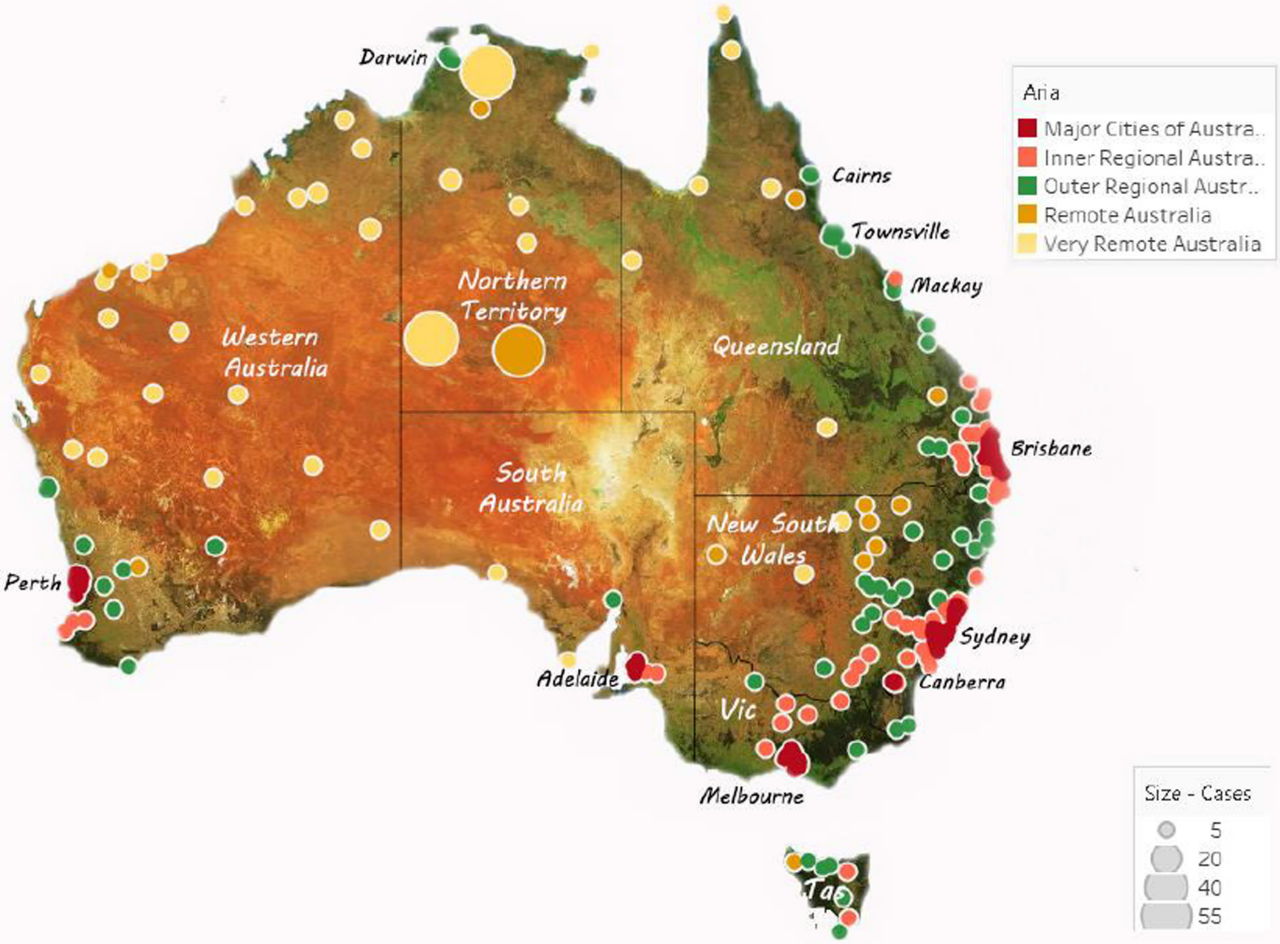
All Aboriginal and/or Torres Strait Islander children with cerebral palsy by Accessibility Remoteness Index Australia (ARIA), birth years 1995 to 2014.

One in five Aboriginal and Torres Strait Islander children/young adults with CP were born or lived in the most socioeconomically advantaged areas (SEIFA quintiles 4 or 5) and two in five in the most socioeconomically disadvantaged areas (quintile 1). Overall, 72% of children lived in urban or regional areas, mostly on the eastern seaboard (Table 2 and Figure 1). Twenty‐eight per cent of children lived in remote and very remote areas, spread across the continent (Table 2 and Figure 1). The largest SEIFA/ARIA classification group, one in five children, was in the most socioeconomically disadvantaged group and lived in remote or very remote Australia. The next largest group comprised those in the most socioeconomically disadvantaged group who were living in major cities (Table 2).

**TABLE 2:** Aboriginal and/or Torres Strait Islander children with cerebral palsy by Socio‐Economic Indexes for Areas (SEIFA) and Accessibility Remoteness Index Australia (ARIA) classifications, 1995–2014.

SEIFA	ARIA	*n* (% of total)
Quintile 1 40.1%	Major Cities of Australia	50 (10.2)
	Inner Regional Australia	26 (5.3)
	Outer Regional Australia	27 (5.5)
	Remote Australia	13 (2.6)
	Very Remote Australia	81 (16.5)
Quintile 2 22.6%	Major Cities of Australia	29 (5.9)
	Inner Regional Australia	18 (3.7)
	Outer Regional Australia	43 (8.8)
	Remote Australia	^a^
	Very Remote Australia	19 (3.9)
Quintile 3 15.9%	Major Cities of Australia	32 (6.5)
	Inner Regional Australia	18 (3.7)
	Outer Regional Australia	16 (3.3)
	Remote Australia	6 (1.2)
	Very Remote Australia	6 (1.2)
Quintile 4 12.2%	Major Cities of Australia	32 (6.5)
	Inner Regional Australia	11 (2.2)
	Outer Regional Australia	6 (1.2)
	Remote Australia	10 (2.0)
	Very Remote Australia	^a^
Quintile 5 9.2%	Major Cities of Australia	33 (6.7)
	Inner Regional Australia	0
	Outer Regional Australia	7 (1.4)
	Remote Australia	0
	Very Remote Australia	5 (1.0)
Total missing		23 (4.5)
Total		491 (100)

^a^ Fewer than five in the cell.

### Birth prevalence

**FIGURE 2:**
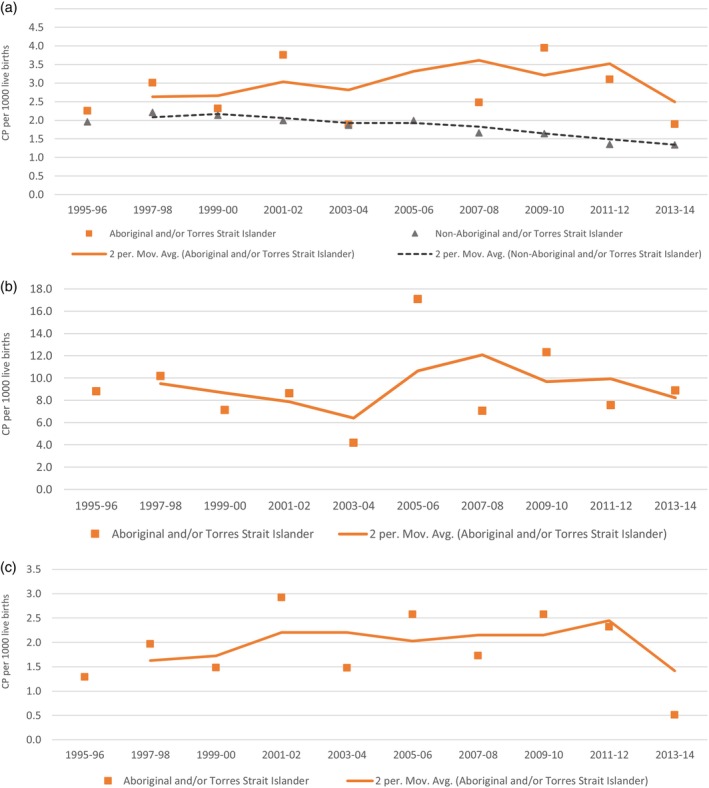
Aboriginal and/or Torres Strait Islander cerebral palsy birth prevalence by 1000 live births, with 2 year moving averages, South Australia, Victoria, and Western Australia combined, 1995 to 2014: (a) prenatal/perinatal cerebral palsy; (b) prenatal/perinatal cerebral palsy, children born <37 weeks; (c) prenatal/perinatal cerebral palsy, children born ≥37 weeks.

Using data from Western Australia, South Australia, and Victoria (*n* = 161), we observed that birth prevalence of prenatally/perinatally acquired CP for Aboriginal and Torres Strait Islander children fluctuated, peaking in the mid‐2000s and decreasing to a low of 1.9 per 1000 live births in the most recent birth years (Figure 2a). The trend was not statistically significant (*p* = 0.09) but since the peak in the mid‐2000s has followed a similar pattern to the decline seen for non‐Indigenous children with CP.

The birth prevalence of CP fluctuated for Aboriginal and Torres Strait Islander children born preterm (*n* = 37) (Figure 2b). There was no trend (*p* = 0.97) and birth prevalence estimates in the most recent birth years (8.9 per 1000 live births) were similar to those of the earliest estimates in the mid‐1990s (8.8 per 1000 live births). Birth prevalence of CP in term‐born infants (*n* = 88) fluctuated (*p* = 0.58) since the early 2000s to a very low point (0.5 per 1000 live births) in the most recent birth years (2013 to 2014) (Figure 2c).

Birth prevalence of CP for Aboriginal and Torres Strait lslander teenage mothers (*n* = 46) declined from 4 per 1000 live births in the mid‐1990s to a low of 1.8 per 1000 live births (most recent birth years), also a non‐significant trend (*p* = 0.81) likely owing to small numbers (Table S1).

There were 26 Aboriginal and Torres Strait Islander children with post‐neonatally acquired CP born in the three states used for the trends analysis. Large fluctuations in prevalence were observed with no trends identified (Table S2).

## DISCUSSION

Aboriginal and Torres Strait Islander peoples are leaders in the concept of inclusion. The centrality of Indigenous culture overshadows impairments such that there is not a word to describe disability in Aboriginal or Torres Strait Islander languages and culture.^5^ Disability is not about personal limitations, but societal responses to these, including the provision of equitable, culturally appropriate services.^5^ However, until now there has been a dearth of evidence on the epidemiology of CP for Aboriginal and Torres Strait Islander people to guide this service provision.

This paper provides a birds‐eye view of CP in Aboriginal and Torres Strait lslander children, young people, and young adults in Australia. The study identified three key results warranting further discussion: (1) one in five Aboriginal and Torres Strait Islander children/young adults lived in socioeconomically disadvantaged remote and very remote areas of Australia; (2) most (72%) of Aboriginal and Torres Strait Islander individuals with CP lived in urban or regional areas; and (3) CP birth prevalence started to decline in the mid‐2000s to a current low of 1.9 per 1000 live births, especially for infants born at term and to teenage mothers.

In Australia, 19% of all Aboriginal and Torres Strait Islander people live in remote or very remote areas compared with 2% of non‐Indigenous people.^3^ Similarly, in this study 20% of Aboriginal and Torres Strait lslander children/young adults with CP lived in remote or very remote areas and were in the lowest SEIFA quintile (our first key result). In our previous research looking at all Australian children with CP and their families there was no difference in SEIFA quintiles compared with the Australian population,^12^ but this is not the case for Aboriginal and Torres Strait lslander children with CP.

It has also been identified that, in general, Australian families do not move house to access services when they have a child with CP, preferring to stay in their own community.^26^ Aboriginal and Torres Strait Islander people are connected to country, and the desire to live on country is strong and is a human right. As such it is essential that children with CP who are living in remote and very remote areas have culturally safe CP‐specific intervention services that are accessible in their place of residence, and additional resourcing for this will be required (as agreed by the National Disability Insurance Scheme Rural and Remote Strategy).^27^ For the 72% of children and young people who are spread widely through urban and regional centres of Australia (our second key result), it is vital that the health and disability workforce are trained in cultural competency and evidence‐based practice, to ensure safe, quality services are experienced by all.

The issue of accessibility of culturally safe services also exists for infants and children who are at risk of neurodevelopmental disability. International guidelines highlight the importance of early detection and intervention for infants with a high risk of CP.^28^, ^29^ Ninety per cent of motor development is reached between the age of 3 and 5 years; therefore early detection and intervention is essential to maximize motor function.^30^ As these guidelines are being implemented in Australia and internationally, it is important that Aboriginal and Torres Strait Islander children, including those living in rural and remote regions, also benefit from this shift in practice. After early identification, access to culturally safe, responsive evidence‐based early intervention that is CP specific, alongside health and well‐being services for children and families, is required. This should be available to all in a wealthy country such as Australia.

Our final key result was that the prevalence of CP had started to decline. This is positive, and there are opportunities to continue monitoring and to build on this momentum. Fifty per cent of Aboriginal and Torres Strait Islander children with CP were born preterm and there was no decline in the birth prevalence of CP for infants born preterm. This lends support to the argument to prioritize Birthing on Country programmes. One programme undertaken in urban Brisbane^31^ reduced preterm births by 40%; if scaled up, to include rural, remote, and very remote areas, this has the potential for huge impact.^32^

There has been a long‐term trend to reduce the number of maternity units (often in rural and remote areas) in many countries.^33^, ^34^ In Australia, this results in pregnant females having to leave home and family weeks before their birth due date, potentially to culturally unsafe maternal and neonatal intensive‐care providers.^35^ Birthing should not be a highly stressful experience; however, rural and remote service delivery favouring policies of centralization of obstetric services has ensured that this is the case for many Aboriginal and Torres Strait Islander people living in rural and remote areas. It is likely that the proportion of families living far away from maternity units will grow, so it is imperative to continually monitor the impact of centralization of obstetric services, while advocating for alternative options for females to deliver their babies in their communities, such as Birthing on Country programmes.^34^, ^36^

Culturally sensitive health promotion programmes focused on healthy pregnancies, such as Mums and Bubs, have been implemented into clinical practice, and are acknowledged as best practice, although they are still yet to be fully funded and provided at scale across the country. Promisingly, there has been an increase in Aboriginal and Torres Strait lslander mothers attending antenatal care in the first trimester (up to 65%) and a decrease in smoking during pregnancy (from 49% to 44% over 10 years).^37^, ^38^ The largest declines in smoking have been among 18‐ to 24‐year‐olds in recent years (2014–2019) in urban areas.^39^ The prevalence of CP among teenage Aboriginal and Torres Strait Islanders seems to be declining. This mirrors the general trend where teenage pregnancy rates have decreased significantly from 70 births per 1000 Indigenous in 2006 to 46 per 1000 in 2017.^40^ Engaging young Aboriginal and Torres Strait Islander people as youth health workers to lead sexual health programmes will help.^41^, ^42^ Opportunities therefore exist to further improve smoking rates and teenage pregnancy, by expanding evidence‐based programmes, particularly into remote areas.

There were some limitations to this study. At the time of data extraction, states with the highest proportions of Aboriginal and Torres Strait Islander people had incomplete ascertainment for their CP registers. It is a priority of the CP registers in these states to become complete, so that trend analyses can be Australia‐wide. The ACPR has historically collected maternal Indigenous status, and only recently started collecting the child's Indigenous status. Consequently, this study may have under‐reported the frequency of CP in Aboriginal and Torres Strait Islander children. We were unable to report ‘age at diagnosis’, as the proportion of missing data was too great. There may also be underestimates of intellectual, speech, and visual impairment in regions where culturally safe services do not currently exist.

The strengths of this study include the fact that it was led by Aboriginal researchers; best practice methodologies were used including a strength‐based perspective and consultation with the ACPR's Community, Aboriginal and Torres Strait Islander Reference Group to gain community input into the development and interpretation of the findings of this study. To date, to our knowledge this is the largest population‐based study of Aboriginal and Torres Strait lslander children with CP conducted in Australia and of First Nations children with CP across the globe.

It is anticipated that key stakeholders such as Aboriginal Community Controlled Health Organisations, the National Disability Insurance Scheme, and health and disability service providers will use this information to advocate for, and allocate appropriate resources to, the expansion of evidence‐based Aboriginal and Torres Strait Islander‐led antenatal care programmes, and services for the health and well‐being needs of children and young adults with CP and their families.

Much work in epidemiology and public health focuses on the presence (or absence) of disease, as we have done here with CP in Aboriginal and Torres Strait Islander peoples. However, to have effective public health in Australia we need to shift these parameters to the Aboriginal and Torres Strait Islander concepts of health: mental, physical, cultural, and spiritual, with land essential to well‐being.^6^ In future research the ACPR group hopes to expand its work with Aboriginal and Torres Strait Islander people to map whether culturally appropriate services are accessible for children with CP, and to determine which health and well‐being outcomes are important and how they should be reported.

## FUNDING INFORMATION

The Cerebral Palsy Alliance Research Foundation through an Early Career Grant for the first author. The Australian Capital Territory, New South Wales and Australian Cerebral Palsy Registers are funded by the Cerebral Palsy Alliance Research Foundation. The Northern Territory Cerebral Palsy Register is funded by Women, Children and Youth, Royal Darwin Hospital. The Queensland Cerebral Palsy Register is funded by Choice, Passion, Life. The South Australian Cerebral Palsy Register is funded by the Women's and Children's Health Network with additional support provided by Novita. The Tasmanian Cerebral Palsy Register is supported by St Giles and the Tasmanian Department of Health. The Victorian Cerebral Palsy Register received funding from the Lorenzo and Pamela Galli Research Trust, the Victorian Department of Health and Human Services, and the Royal Children's Hospital Foundation, and infrastructure support was provided by the Victorian Government's Operational Infrastructure Support Program. The Western Australian Register of Developmental Anomalies ‐ Cerebral Palsy is funded by Department of Health Western Australia. HSS received salary support through a National Health and Medical Research Council of Australia Early Career Fellowship (1144566) and Australasian Cerebral Palsy Clinical Trials Network.

## Supporting information


**Table S1:** Aboriginal and/or Torres Strait Islander pre/perinatal cerebral palsy birth prevalence by 1000 live births

**Table S2:** Aboriginal and/or Torres Strait Islander post‐neonatally acquired cerebral palsy birth prevalence by 10 000 live births

## Data Availability

Research data are not shared.
