# Supplementary material for: Prevalence and trends for Aboriginal and Torres Strait Islander children living with cerebral palsy: A birds‐eye view
Source: Dev Med Child Neurol. 2023 May 5;65(11):1475–85. doi: 10.1111/dmcn.15617 (PMC10952932; doi:10.1111/dmcn.15617)
Supplement: Supplementary file 1 — Table S1: Aboriginal and/or Torres Strait Islander pre/perinatal cerebral palsy birth prevalence by 1000 live births [file DMCN-65-1475-s002.docx]

**Table S1 Aboriginal and/or Torres Strait Islander pre/perinatal cerebral palsy birth prevalence by 1000 Live Births with 95% Confidence Intervals (CI) South Australia Victoria and Western Australia**

|  | **Live births** | **Cerebral palsy n** | | **Prevalenceper 1000 LB (95%CI)** | | |
| --- | --- | --- | --- | --- | --- | --- |
| 1995-96 | 4,437 | 10 | | 2.3 (1.2, 4.1) | | |
| 1997-98 | 4657 | 14 | | 3.0 (1.8, 5.0) | | |
| 1999-00 | 4,750 | 11 | | 2.3 (1.3, 4.1) | | |
| 2001-02 | 4,797 | 18 | | 3.8 (2.4, 5.9) | | |
| 2003-04 | 4,781 | 9 | | 1.9 (1.0. 3.6) | | |
| 2005-06 | 5,473 | 26 | | 4.8 (3.2, 7.0) | | |
| 2007-08 | 6,060 | 15 | | 2.5 (1.5, 4.1) | | |
| 2009-10 | 6,334 | 25 | | 3.9 (2.7, 5.8) | | |
| 2011-12 | 6,460 | 20 | | 3.1 (2.0, 4.8) | | |
| 2013-14 | 6,880 | 13 | | 1.9 (1.1, 3.2) | | |
| Trend *p*=0.09 | | | | | | |
|  | | | | | | |
|  | **Born <37 weeks Gestational Age** | | | **Born ≥37 weeks Gestational Age** | | |
|  | **Live births** | **Cerebral palsy n** | **Prevalence**  **(95% CI)** | **Live births** | **Cerebral palsy n** | **Prevalence**  **(95% CI)** |
| 1995-96 | 567 | 5 | 8.8 (3.8, 20.5) | 3,866 | 5 | 1.3 (0.5, 3.0) |
| 1997-98 | 589 | 6 | 10.2 (4.7, 22.0) | 4,068 | 8 | 2.0 (1.0, 3.9) |
| 1999-00 | 701 | 5 | 7.1 (3.1, 16.6) | 4,048 | 6 | 1.5 (0.7, 3.2) |
| 2001-02 | 696 | 6 | 8.6 (4.0, 18.7) | 4,101 | 12 | 2.9 (1.7, 5.1) |
| 2003-04 | 717 | @ | 4.2 (1.4, 12.2) | 4,063 | 6 | 1.5 (0.7, 3.2) |
| 2005-06 | 819 | 14 | 17.1 (10.2, 28.5) | 4,654 | 12 | 2.6 (1.5, 4.5) |
| 2007-08 | 849 | 6 | 7.1 (3.2, 15.3) | 5,211 | 9 | 1.7 (0.9, 3.2) |
| 2009-10 | 894 | 11 | 12.3 (6.9, 21.9) | 5,436 | 14 | 2.6 (1.5, 4.3) |
| 2011-12 | 924 | 7 | 7.6 (3.7, 15.6) | 5,604 | 13 | 2.3 (1.3, 3.9) |
| 2013-14 | 1,012 | 9 | 8.9 (4.7, 16.8) | 5,868 | @ | 0.5 (0.2, 1.5) |
| Trend p=0.97 *p*=0.58 | | | | | | |
|  |  | | | | | |
| **Mothers age at birth of child with cerebral palsy <20 years ≥20 years** | | | | | | |
|  | **Live births** | **Cerebral palsy n** | **Prevalence**  **(95% CI)** | **Live births** | **Cerebral palsy n** | **Prevalence**  **(95% CI)** |
| 1995-96 | 1,009 | @ | 4.0 (1.5, 10.1) | 3,428 | 6 | 1.8 (0.8, 3.8) |
| 1997-98 | 1,031 | @ | 3.9 (1.5, 9.9) | 3,656 | 10 | 2.7 (1.4, 5.0) |
| 1999-00 | 1,066 | @ | 3.8 (1.5, 9.6) | 3,684 | 7 | 1.9 (0.9, 3.9) |
| 2001-02 | 1,071 | @ | 3.7 (1.4, 9.5) | 3,726 | 14 | 3.8 (2.2, 6.2) |
| 2003-04 | 1,122 | @ | 3.6 (1.4, 9.1) | 3,659 | 5 | 1.4 (0.5, 3.2) |
| 2005-06 | 1,264 | 10 | 7.9 (4.3, 14.5) | 4,209 | 14 | 3.3 (2.0, 5.6) |
| 2007-08 | 1,236 | @ | 3.2 (1.3, 8.3) | 4,824 | 11 | 2.3 (1.3, 4.1) |
| 2009-10 | 1,240 | 5 | 4.0 (1.7, 9.4) | 5,089 | 19 | 3.7 (2.4, 5.8) |
| 2011-12 | 1,184 | 5 | 4.2 (1.8, 9.8) | 5,344 | 14 | 2.6 (1.6, 4.4) |
| 2013-14 | 1,123 | @ | 1.8 (0.5, 6.4) | 5,757 | 10 | 1.7 (0.9, 3.1) |
| Trend |  |  | *p*=0.81 |  |  | *p*=0.80 |

@ n<5
