# Supplementary material for: Prevalence and trends for Aboriginal and Torres Strait Islander children living with cerebral palsy: A birds‐eye view
Source: Dev Med Child Neurol. 2023 May 5;65(11):1475–85. doi: 10.1111/dmcn.15617 (PMC10952932; doi:10.1111/dmcn.15617)
Supplement: Supplementary file 2 — Table S2: Aboriginal and/or Torres Strait Islander post‐neonatally acquired cerebral palsy birth prevalence by 10 000 live births [file DMCN-65-1475-s001.docx]

**Table S2 Aboriginal and/or Torres Strait Islander post-neonatally acquired cerebral palsy birth prevalence by 10,000 LB with 95% Confidence Intervals South Australia Victoria and Western Australia**

|  | | | |
| --- | --- | --- | --- |
|  | **Live births** | **Cerebral palsy n** | **Prevalence per 10,000 LB (95%CI)** |
| 1995-96 | 4,437 | @ | 6.8 (2.2, 19.9) |
| 1997-98 | 4,657 | @ | 4.3 (1.1, 15.6) |
| 1999-00 | 4,750 | 5 | 10.5 (4.5, 24.6) |
| 2001-02 | 4,797 | @ | 2.1 (0.4, 11.8) |
| 2003-04 | 4,781 | @ | 2.1 (0.4, 11.8) |
| 2005-06 | 5,473 | @ | 5.5 (1.9, 16.1) |
| 2007-08 | 6,060 | @ | 3.3 (0.9, 12.0) |
| 2009-10 | 6,334 | 5 | 7.9 (3.4, 18.5) |
| 2011-12 | 6,460 | @ | 1.5 (0.3, 8.8) |
| 2013-14 | 6,880 | @ | 4.4 (1.5, 12.8) |
|  |  |  | *p*=0.80 |
| @ n<5 | | | |
